# Medical Appointments

**Published:** 1847-11

**Authors:** 


In the Medical Examiner for October we find the following list of appointments recently made in the medical institutions in this country. Some time since, on the authority of an eastern publication, we announced the election of Professor Henry, of Princeton, to the chair of Chemistry in the University of Pennsylvania, but the announcement seems to have been incorrect. A better appointment, we may remark in passing, it would not have been in the power of the trustees easily to have made.

Prof. Henry would confer distinction upon any institution of the country. Dr. Rogers, too, is well known as an able practical chemist and eloquent lecturer. Those who were connected with him in the Cincinnati Medical College speak of him in most cordial terms, and he carries with him into his new sphere of labor the admiration of a large body of pupils.

*University of Pennsylvania.*—James B. Rogers, M.D., Professor of Chemistry, vice Professor Hare, resigned.

*Ohio Medical College.*—Professor L. M. Lawson, of the University of Transylvania, Professsor of Materia Medica, vice Prof. Harrison, transferred to the chair of Theory and Practice of Medicine.

*Hampden Sidney College.*—Charles Bell Gibson, M.D., Professor of Surgery, vice Prof. Warner, deceased.

*University of New York.*—Prof. S. H. Dickson, of the Medical College of South Carolina, Professor of Theory and Practice, vice Prof. Revere, deceased.

*College of Physicians and Surgeons of the City of New York.*—Prof. Alonzo Clark, of Pittsfield, Lecturer on Physiology and Pathology.

*Medical College of South Carolina.*—Dr. Bellinger, Professor of Surgery, in the place of Prof. Geddings, transferred to the chair of Practice, vice Dr. Dickson, resigned.

*Philadelphia College of Medicine.*—Henry Gibbons, M.D., Professor of Institutes and Practice of Medicine, vice Prof. Thos. D. Mitchell, resigned; D. P. Gardiner, M.D., Professor of Chemistry, vice Prof. Allen, resigned; Louis H. Beatty, M.D., Professor of Obstetrics.

*Hampden Sidney College and University of Virginia.*—Prof. Cabell, whose appointment to the chair of Surgery in Hampden Sidney College was recently announced, declines the appointment, and retains his connection with the University of Virginia.

